# Changes in the Gut Microbiota of Rats in High-Altitude Hypoxic Environments

**DOI:** 10.1128/spectrum.01626-22

**Published:** 2022-10-27

**Authors:** Xue Bai, Guiqin Liu, Jianxin Yang, Junbo Zhu, Qian Wang, Yang Zhou, Wenqi Gu, Linli La, Xiangyang Li

**Affiliations:** a Research Center for High Altitude Medicine, Qinghai University Medical College, Xining City, China; b State Key Laboratory of Plateau Ecology and Agriculture, Qinghai University, Xining City, China; c Medical College, Qinghai University Medical College, Xining City, China; U.S. Food and Drug Administration, National Center for Toxicological Research

**Keywords:** 16S rRNA gene, diversity, fecal microbiota transplantation, gut microbiota, high-altitude hypoxia, structure

## Abstract

This study was conducted to investigate the effects of high-altitude hypoxic environments on the gut microbiota. Male Sprague-Dawley rats were randomly divided into three groups, namely, the plain, moderate-altitude hypoxic, and high-altitude hypoxic groups. On the 3rd, 7th, 15th, and 30th days of exposure, fecal samples were collected and analyzed via 16S rRNA gene sequencing technology. Fecal microbiota transplantation (FMT) experiments were also performed. The results showed significant differences between the gut microbiota structure and diversity of rats in the high-altitude hypoxic group and those of rats in the other groups. Further, compared with that of rats in the plain group, the gut microbiota of rats in the two hypoxic groups showed the most significant changes on day 7. Furthermore, the gut microbiota of the rats in the FMT groups exhibited changes and became increasingly similar to those of the rats in the hypoxic groups. We also identified the phylum *Firmicutes*, genus *Akkermansia*, and genus *Lactobacillus* as the core microbiota under hypoxic conditions. Phenotypic analysis indicated a decrease in the proportion of aerobic bacteria and an increase in that of anaerobic bacteria, possibly owing to the high-altitude hypoxic environment. Additionally, functional analysis showed significant differences between the different groups with respect to different metabolic pathways, including carbohydrate metabolism, energy metabolism, glycan biosynthesis, and metabolism. These findings indicated significant changes in gut microbiota structure and diversity under high-altitude hypoxia, establishing a foundation for further research on the pathogenesis and development of diseases, as well as drug metabolism, under high-altitude hypoxia.

**IMPORTANCE** In this study, we investigated the effects of high-altitude hypoxic environments with low oxygen levels on the gut microbiota characteristics of rats. We observed that high-altitude hypoxia is an important environmental factor that can affect gut microbiota structure and diversity, thereby affecting homeostasis in the host intestinal environment. These findings provide a basis for further studies on disease initiation and development, as well as drug metabolism, in high-altitude hypoxic environments.

## INTRODUCTION

High-altitude hypoxia is a unique ecological environment characterized by low oxygen levels, low pressure, and high radiation, with low oxygen levels being the main factor that affects humans (1). Hypoxia-induced changes in bodily functions, metabolism, and structure are unique pathophysiological responses to plateau-related diseases (2–4). Some pathological changes can cause intestinal problems, including intestinal mucosal damage, atrophy, and barrier dysfunction, leading to changes in intestinal microecology under high-altitude hypoxia (5, 6).

Trillions of microbes inhabit the intestines; collectively, they are known as the gut microbiota, which constitutes a complex ecological community that influences normal physiology and disease susceptibility (7). The diversity and composition of the gut microbiota are affected by various factors, including host genotype, diet, and environment (8). Under pathological conditions, the dynamic microbial community undergoes certain changes. Microbiome studies have attracted considerable attention in recent years, with the main focus being chronic metabolic diseases (such as diabetes and obesity) and inflammatory bowel diseases (such as Crohn’s disease and ulcerative colitis) (9). Notably, fecal microbiota transplantation (FMT) has been used for the treatment of intestinal and extra-intestinal diseases, with various degrees of success (10). Further, numerous studies have demonstrated that the use of FMT to treat some diseases can be realized by transferring feces from healthy donors to patients with gut microbial imbalances (11). Similarly, transferring disturbed gut microbes to receptors with normal gut microbiota structure can reshape the gut microbiota of these normal receptors, causing them to exhibit the intestinal microbial composition and diversity of the donor (12). However, the extent to which FMT brings about changes in the gut microbiota of recipients remains unclear. Therefore, further research is required to determine whether the transplantation of disturbed gut microbiota can modify gastrointestinal microbial imprints and subsequently lead to changes in gut function.

Some researchers have emphasized that changes in intestinal microecology are closely related to high-altitude hypoxic environments. Zhang et al. (13) analyzed the changes in the gut microbial community of mice by simulating an altitude of 5 km. Their results showed a significant difference in β-diversity between the 5-km-altitude group and the controls, whereas no significant differences were observed with respect to α-diversity. Ma et al. (14) also showed that the structure and diversity of the gut microbial community in rats changed under hypoxia. Additionally, Jia et al. (15) reported that the low-oxygen environment of a plateau has a profound impact on gut microbiota structure. To date, studies on the relationship between changes in gut microbiota composition and diversity and changes in altitude in real plateau hypoxic environments are limited. Further, the combined effect of various hypoxic factors (such as acute hypoxia, chronic hypoxia, moderate altitude, and high altitude) on gut microbiota has not yet been investigated. Thus, further studies are needed in this regard. In this study, 16S rRNA gene sequencing was performed to explore the effects of different altitudes and hypoxia treatment times on the structure and diversity of the gut microbiota of rats. FMT experiments were also performed to further clarify the relationship between gut microbiota and high-altitude hypoxic environments. We demonstrated that exposure to high-altitude hypoxic environments can bring about significant changes in gut microbiota structure and diversity.

## RESULTS

### Variations in gut microbiota diversity following exposure to high-altitude hypoxic environments.

**FIG 1:**
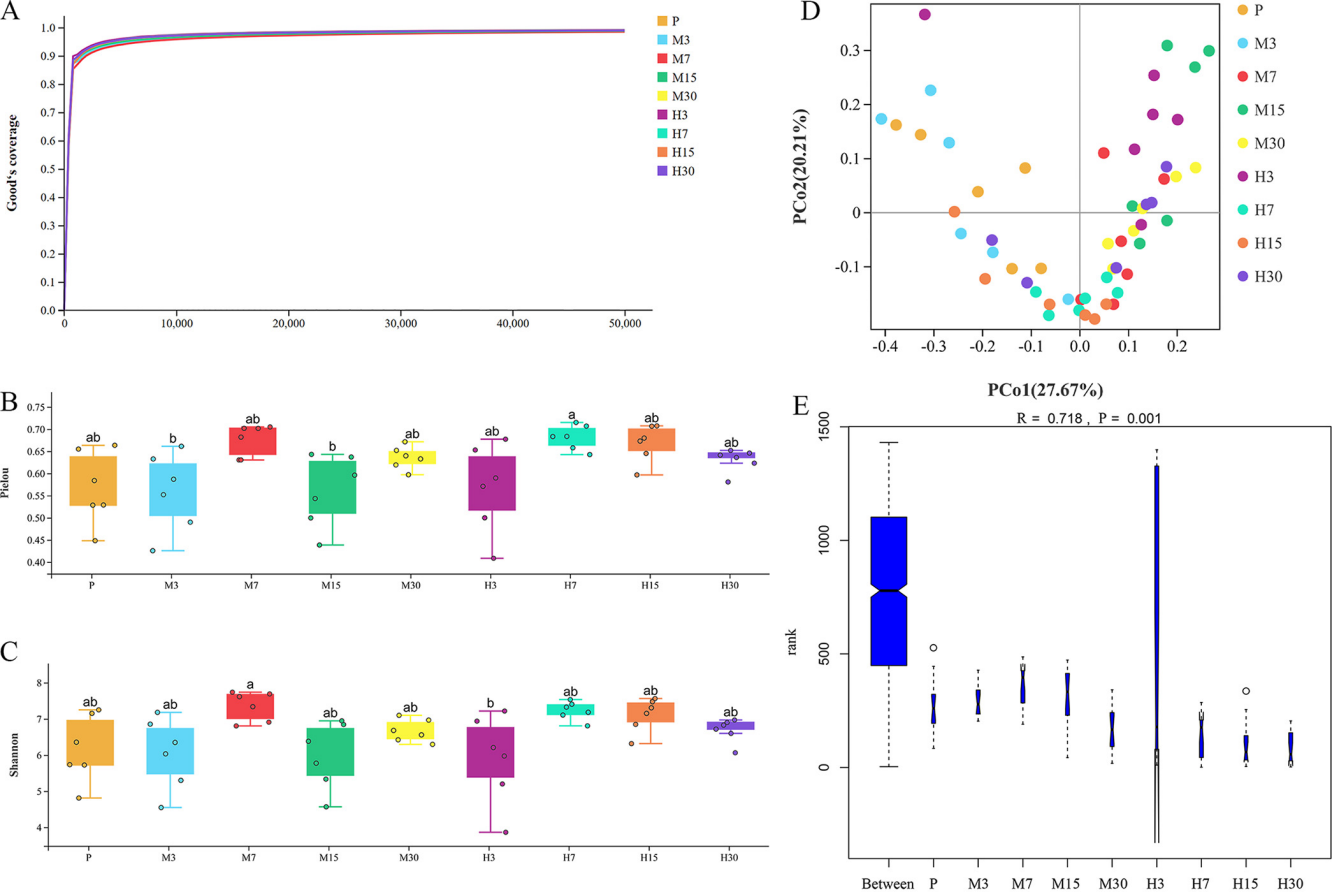
Diversity analysis of gut microbiota of rats. (A) Dilution curve of Good’s coverage; (B) Pielou index; (C) Shannon index; (D) principal-coordinate analysis with weighted UniFrac distance; (E) ANOSIM for weighted UniFrac. P, plain group; M3, M7, M15, and M30, 3rd, 7th, 15th, and 30th days of the moderate-altitude hypoxic group; H3, H7, H15, and H30, 3rd, 7th, 15th, and 30th days of the high-altitude hypoxic group. Means with same box followed by different superscript letters differ at a *P *value of <0.05.

Rarefaction curves showed that all the samples reached a plateau (Fig. 1A), indicating that our sequencing depth was sufficient for investigating the microbial communities in the collected fecal samples. Significant differences (*P < *0.05) in microbial community richness (Pielou index) and diversity (Shannon index) were observed across samples at different altitudes and hypoxia treatment times (Fig. 1B and C). Considering all the experimental groups, the M and H groups (see Materials and Methods) showed the highest diversity indices on day 7.

Principal-coordinate analysis (PCoA) based on weighted UniFrac distance metrics together with analysis of similarities (ANOSIM) was performed (Fig. 1D and E). The results showed that gut microbiota composition was affected by different altitudes, whereas hypoxia treatment times had a limited effect.

### Taxonomy-based comparisons of gut microbiota at the phylum and genus levels in high-altitude hypoxic environments.

**FIG 2:**
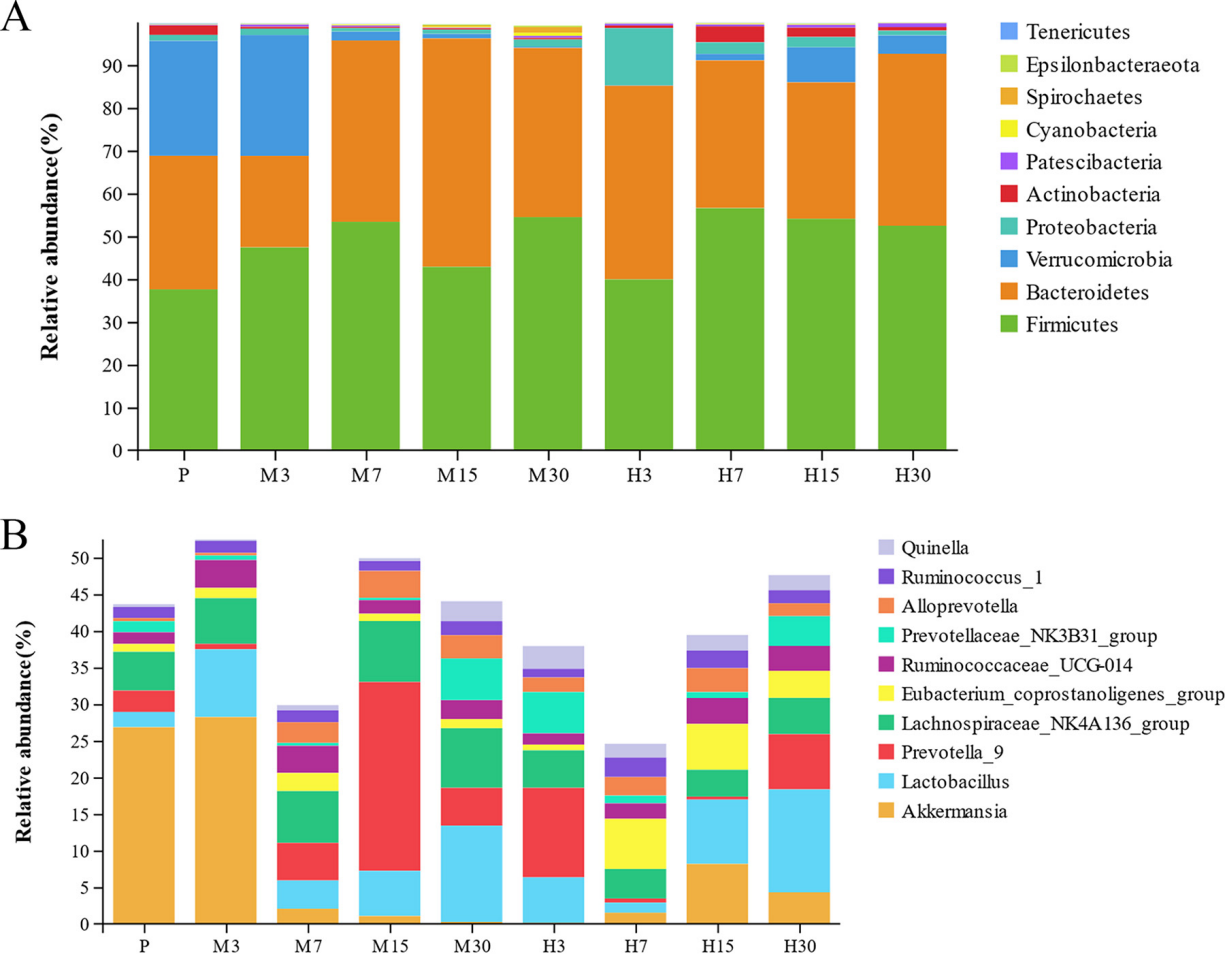
Histogram of community structure of gut microbiota in rats at the phylum and genus levels. (A) Phylum level; (B) genus level.

Overall gut microbiota compositions of each group at the phylum and genus levels are shown in Fig. 2A and B. At the phylum level, the predominant phylum was *Firmicutes*, followed by *Bacteroidetes*. Other relatively abundant phyla included *Verrucomicrobia*, *Proteobacteria*, and *Actinobacteria*. Further, the relative abundance of *Firmicutes* in the M7 and H7 groups was higher than in all other groups. Our results also indicated that *Verrucomicrobia* was the dominant phylum in the P and M3 groups. In the M7 group, the *Proteobacteria* had lower relative abundance than in other groups. In the H7 group, the *Actinobacteria* had the higher relative abundance than in other groups.

Considering all the samples, the top 10 most abundant genera were *Akkermansia*, *Lactobacillus*, *Prevotella_*9, *Lachnospiraceae_NK4A136_*group, *Eubacterium_coprostanoligenes_*group, *Ruminococcaceae_UCG-*014, *Prevotellaceae_NK3B31_*group, *Alloprevotella*, *Ruminococcus_*1, and *Quinella*. Specifically, the relative abundance of *Akkermansia* was higher in the P and M3 groups than in all other groups. *Lactobacillus* showed higher relative abundance in the M30 and H30 groups than in other groups, and the relative abundance of *Prevotella_*9 in the H7 group was much lower than those in the other groups, especially that corresponding to the P group.

### Differences in the gut microbiota composition at different altitudes.

**FIG 3:**
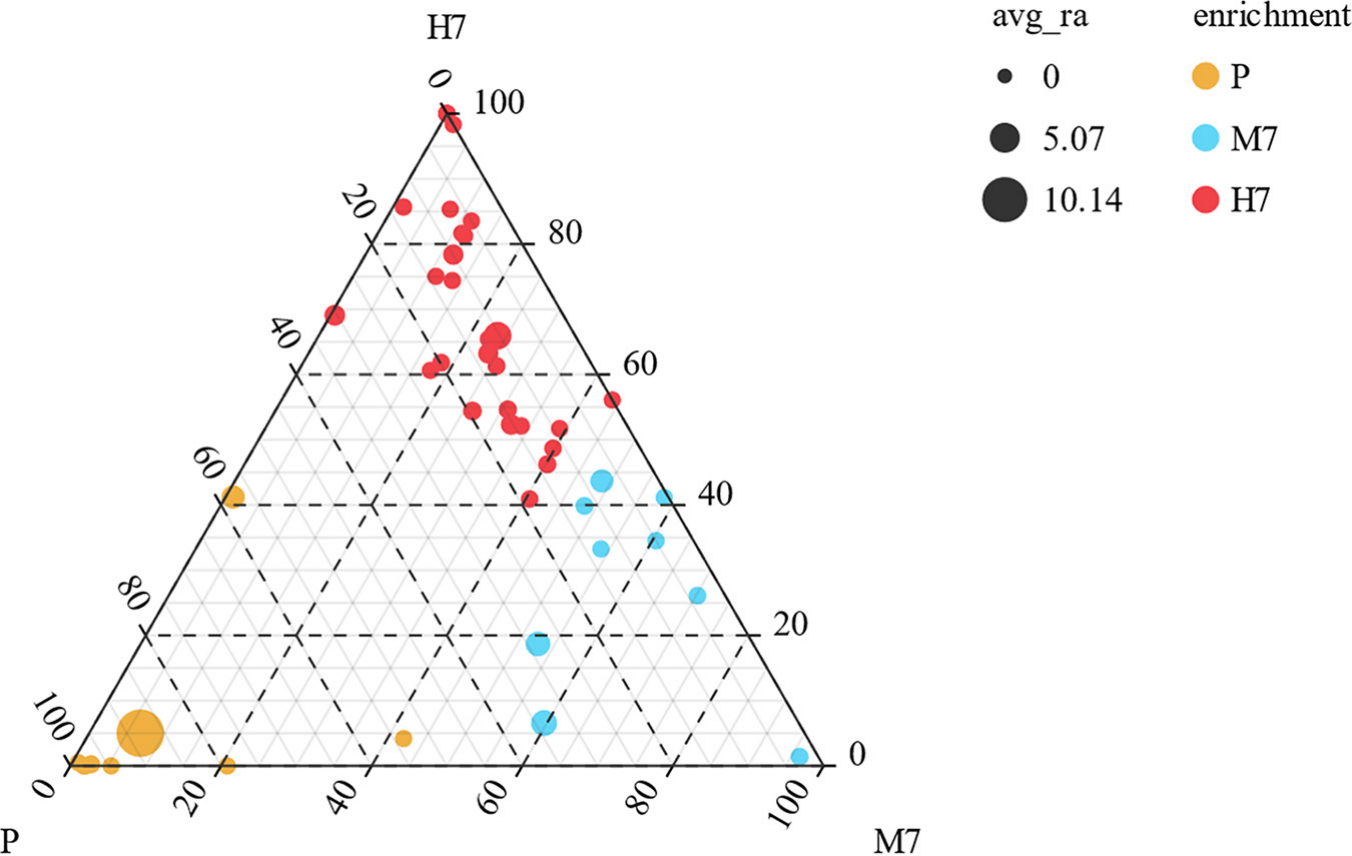
Analysis of differences in the microbiota among different altitudes.

The analysis of differences in taxon abundance was performed using ternary diagrams to explore the differences in gut microbiota composition at different altitudes. Thus, differences in gut microbiota among the different groups were identified. The M7 group showed higher relative abundances of *Lactobacillus*, *Prevotella_*9, *Alloprevotella*, *Ruminococcaceae_UCG-*009, *Ruminococcus_*2, *Lachnospiraceae_UCG-*001, *Negativibacillus*, *Butyricicoccus*, and *Caproiciproducens* than the other groups. Further, the H7 group showed higher relative abundances of *Eubacterium_coprostanoligenes_*group, *Quinella*, *Bifidobacterium*, *Romboutsia*, *Ruminococcaceae_NK4A214_*group, *Stenotrophomonas*, *Ruminiclostridium_*9, *Lachnoclostridium*, *Ruminococcaceae_UCG-*010, *Ruminococcaceae_UCG-*004, *Anaerovorax*, *Enterorhabdus*, *Coriobacteriaceae_UCG-*002, *Anaerotruncus*, *Sphingobacterium*, and *Odoribacter* than the other groups. Interestingly, the genera *Myroides*, *Alicycliphilus*, *Xanthobacter*, *Achromobacter*, *Bosea*, and *Cellulosimicrobium* were endemic to the H7 group. Possibly, these differences in gut microbial composition for the different altitude groups were closely related to the plateau hypoxic environment (Fig. 3).

### Differences in the gut microbiota composition following different hypoxia treatment times.

**FIG 4:**
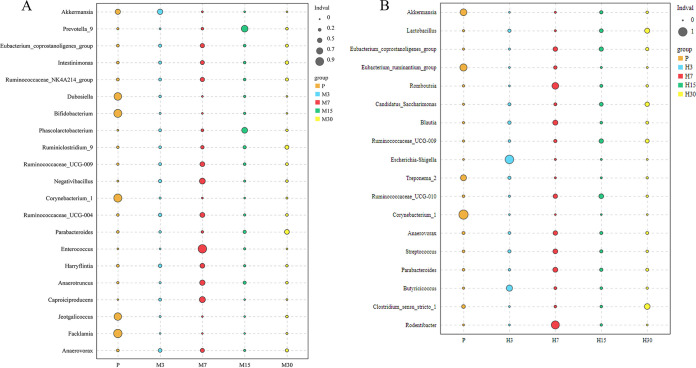
Analysis of differences in the microbiota among different high-hypoxic altitude treatment times. (A) Moderate-altitude hypoxic groups; (B) high-altitude hypoxic groups.

The taxonomic abundances of the gut microbiota of rats in different groups at the same altitude and different treatment times were compared using a bubble chart. The results showed that the M7 group had higher relative abundances of *Enterococcus*, *Caproiciproducens*, *Negativibacillus*, *Anaerotruncus*, *Anaerotruncus*, *Ruminococcaceae*_*UCG*-009, *Harryflintia*, *Ruminococcaceae*_*NK4A214*_group, *Eubacterium*_*coprostanoligenes*_group, *Anaerovorax*, and *Intestinimonas* than the other groups, while the relative abundances of *Facklamia* and *Jeotgalicoccus* were lower in the M7 group than in the other groups (Fig. 4A).

The gut microbiota of the H7 group showed several changes as well as several other types of microbial species compared with the other groups. Specifically, this group showed greater proportions of *Rodentibacter*, *Romboutsia*, *Coriobacteriaceae_UCG-*002, *Adlercreutzia*, *Butyrivibrio*, *Bifidobacterium*, *Bacteroides*, *Blautia*, *Ruminiclostridium*, *Eubacterium_brachy_*group, *Parabacteroides*, *Lachnospira*, *Parasutterella*, *Lachnoclostridium*, *Enterorhabdus*, *Butyricimonas*, and *Angelakisella* than other groups. Only the proportions of *Odoribacter*, *Dubosiella*, *Veillonella*, and *Enterococcus* in the H7 group were lower than those in the other groups (Fig. 4B).

### Functional alteration of the gut microbiota in a high-altitude hypoxic environment.

**FIG 5:**
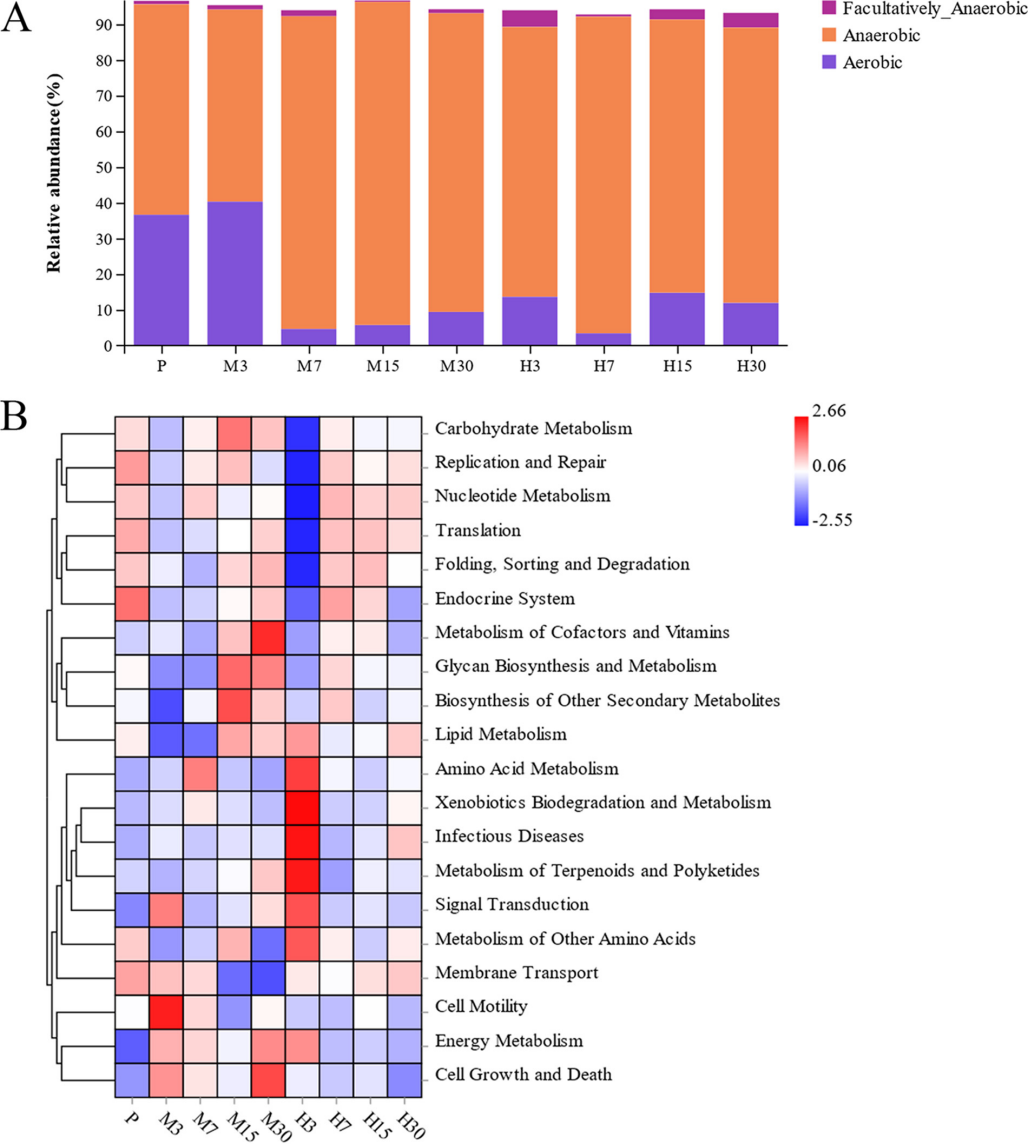
Functional analysis of the gut microbiota in each group of rats. (A) BugBase functional analysis; (B) Tax4Fun functional prediction analysis.

The distribution of the relative abundances of different phyla and genera in the gut microbiota in each group based on BugBase analysis is shown in Fig. 5A. In particular, compared to that in the P group, the proportion of aerobic bacteria in the hypoxic group was reduced. Further, the M7 and H7 groups showed the lowest abundances in this regard. Compared with that in the P group, the relative abundance of anaerobic bacteria was greatly increased in the hypoxic groups. Based on these findings, we hypothesized that a correlation possibly exists between a high-altitude hypoxic environment and a decrease and increase in the proportion of aerobic and anaerobic bacteria, respectively.

The results obtained following analysis using Taxt4Fun are shown in Fig. 5B. From this figure, it is evident that differences existed between the different groups with respect to pathways, such as carbohydrate metabolism, energy metabolism, glycan biosynthesis, and metabolism. The predictive function of the gut microbiota of rats in the M groups was mainly focused on cellular processes and drug-related metabolisms, such as the regulation of basic transcription factors, protein transport, and regulation of drug metabolism enzymes. In addition, the gut microbiota of rats in the H groups was predominantly associated with functions, such as genetic information processing and drug-related metabolism, including fatty acid biosynthesis, cytochrome P450 xenobiotic metabolism, and pentose phosphate pathways.

### Hypoxia-to-normal FMT triggered changes in gut microbial composition.

**FIG 6:**
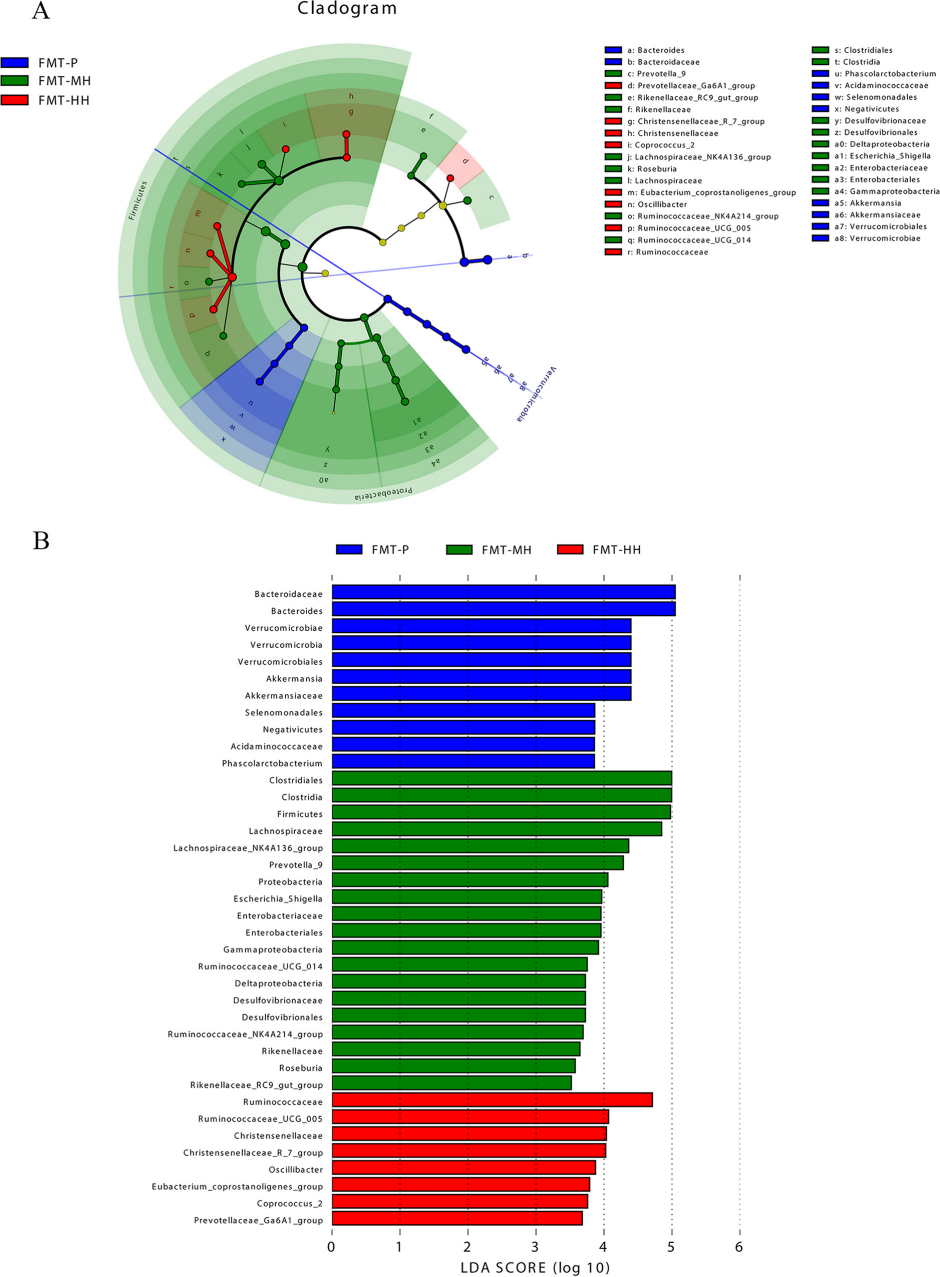
Histogram of community structure of gut microbiota in rats at the phylum and genus levels. (A) Phylum level; (B) genus level. FMT-P, normal rats receiving autoclaved saline; FMT-MH, normal rats receiving fecal suspension from the 7th-day moderate-altitude hypoxic donor rats; FMT-HH, normal rats receiving fecal suspension from the 7th-day high-altitude hypoxic donor rats.

The differences in the gut microbiota were also compared via linear discriminant analysis effect size (LEfSe) analysis according to a linear discriminant analysis score of ≥3.5 (Fig. 6A), and a cladogram was used to illustrate the relationships between different taxa (Fig. 6B). *Verrucomicrobia* was the dominant phylum in the FMT-P group. *Proteobacteria* and *Bacteroidetes* were the dominant phyla in the FMT-MH group. *Phascolarctobacterium*, *Bacteroides*, *Akkermansia*, and *Verrucomicrobiales* were the predominant genera in the FMT-P group. *Escherichia*_*Shigella*, *Ruminococcaceae_NK4A214*_group, *Roseburia*, *Prevotella*_9, *Ruminococcaceae_UCG*-014, *Rikenellaceae_RC9*_gut_group, and *Lachnospiraceae_NK4A136*_group were the predominant genera in the FMT-MH group. *Coprococcus*_2, *Oscillibacter*, *Eubacterium*_*coprostanoligenes*_group, *Christensenellaceae*_R_7_group, *Ruminococcaceae_UCG*-005, and *Prevotellaceae_Ga6A1*_group were the predominant genera in the FMT-HH group.

### FMT-related associations with gut microbial changes.

**FIG 7:**
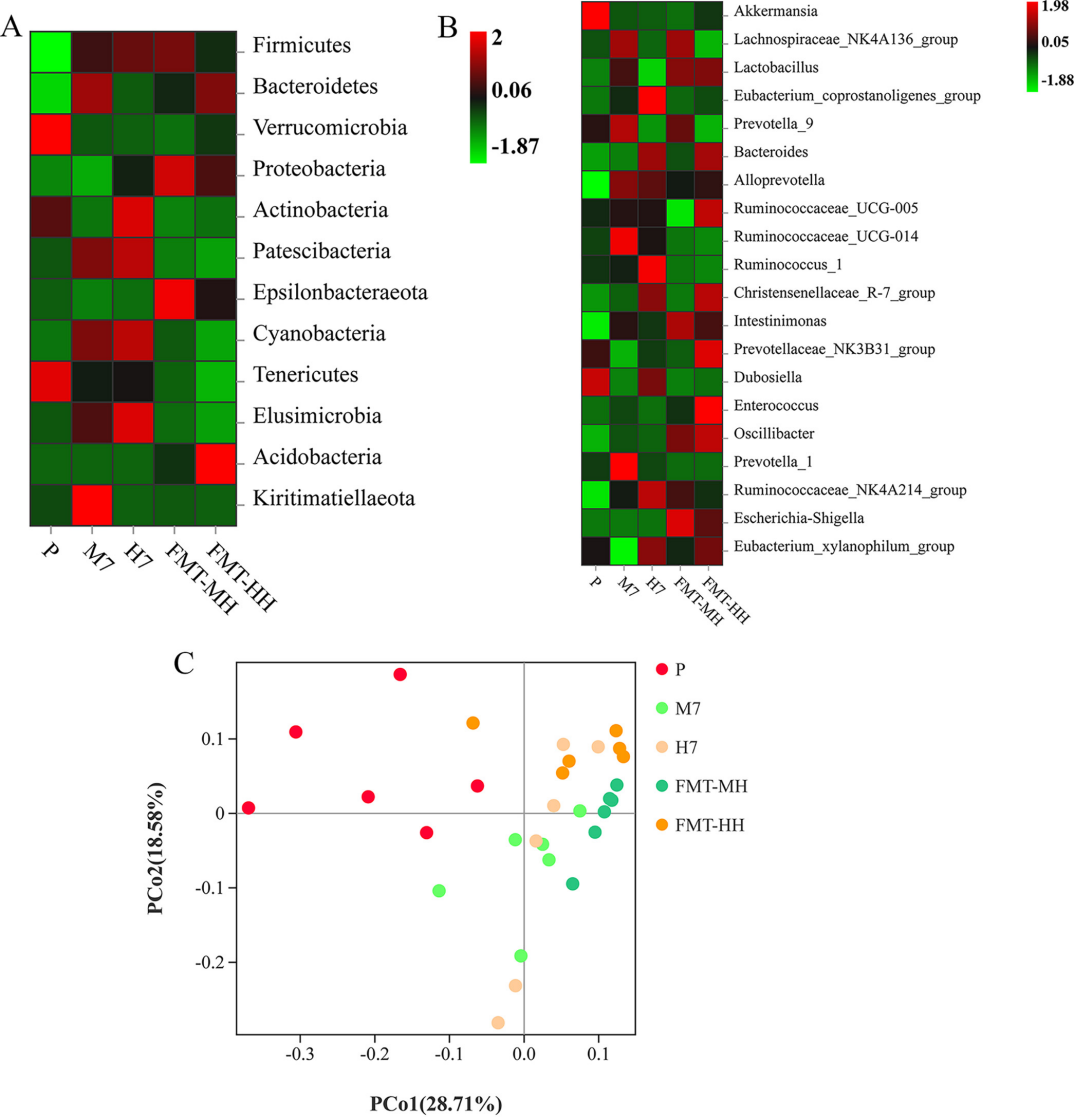
FMT-related associations with gut microbial changes. (A) Phylum level; (B) genus level; (C) principal-coordinate analysis with weighted UniFrac distance.

To further investigate FMT-related associations, bacteria that significantly changed in the FMT recipient groups were selected for correlation analysis, as they were more likely to introduce host metabolic alterations. In this study, we mainly focused on one phylum (*Actinobacteria*) and six genera (*Lachnospiraceae_NK4A136*_group, *Prevotella_*9, *Bacteroides*, *Ruminococcaceae_UCG*-014, *Christensenellaceae_R-7*_group, and *Dubosiella*) that changed dramatically in the FMT and hypoxic groups. Interestingly, similar trends were observed among the groups (Fig. 7A and B).

Notably, samples from the M7 and FMT-MH groups showed an aggregation trend similar to that of samples from the H7 and FMT-HH groups (Fig. 7C). This observation indicated that the colonization of fecal bacteria at different altitudes had a greater impact on the structure and diversity of the gut microbiota of rats.

## DISCUSSION

In this study, we used rats as animal models to evaluate the effect of changes in geographical altitude on the gut microbiota. The experimental conditions were strictly controlled to avoid the influence of other factors, such as genetic background and dietary habits (16, 17). Specifically, in this study, the rats were housed at different hypoxic altitudes, and at different time points, fecal samples were collected for 16S rRNA gene sequencing. Thereafter, the gut microbiota compositions of the hypoxia rats were compared with that of the plain rats. Moreover, an FMT experiment was performed to further confirm whether plateau hypoxia could lead to changes in the structure and diversity of the gut microbiota of rats.

We also compared the gut microbiota of the different groups at different taxonomic levels. We observed that the gut microbiota communities of rats housed on the plain were considerably different from those of hypoxia-treated rats. Additionally, in this study, we not only evaluated the influence of hypoxia treatment on microbial composition but also showed the effect of treatment time (in this case, hypoxia exposure time) on microbial composition. Interestingly, the gut microbiota of rats in the M and H groups showed the most significant and lasting changes on day 7, suggesting that the influence of hypoxia treatment time on microbiota is rapid, persistent, and directional. Compared with that of rats in the P group, the gut microbiota of rats in the FMT groups changed and became increasingly similar to those of rats in the hypoxic groups. This observation indicated that plateau hypoxia had a significant impact on gut microbiota composition, and the changes in the composition might be related to the plateau hypoxia adaptation process (18).

Statistical analysis showed significant differences in the overall bacterial diversity of the gut microbiota of rats, inconsistent with the results of a previous study. Using hypobaric-chamber housed mice, Zhang et al. (13) noted no statistically significant differences in α-diversity relative to the control group. Additionally, Li and Zhao (17) reported no significant differences in the diversity of the gut microbiota of humans belonging to two ethnic groups living at high and low altitudes. These differences between the previously reported results and our results might be related to the differences in the hypoxia treatment methods and research objects used. For example, this study focused on a real plateau hypoxic environment rather than a simulated hypoxia environment.

Oxygen plays a key role in maintaining the balance between aerobic and anaerobic conditions in the intestinal epithelium (19). The highly anaerobic state of intestinal epithelial cells reduces the number of aerobic bacteria and favors the growth of anaerobic bacteria in the intestinal lumen microenvironment (20, 21). *Lactobacillus* (phylum *Firmicutes*) is an anaerobic bacterium whose increased relative abundance contributes to the protection of the intestinal mucosal barrier and improves intestinal function (22). Our results indicated that *Lactobacillus* could have a stronger ability to adapt to a high-altitude hypoxic environment. In line with our observations, Wang et al. (23) reported that adaptation to a low-oxygen environment could contribute to the ability of *Lactobacillus* to colonize the host intestinal tract. Our results also indicated that the high-altitude hypoxic environment decreased the relative abundance of *Akkermansia*. Deficiency of *Akkermansia* impairs intestinal integrity and increases intestinal leakage, ultimately leading to inflammation and insulin resistance, among other effects (24, 25). Zhou (26) reported that the relative abundance of *Akkermansia* as a probiotic was negatively correlated with inflammation and metabolic disorders. *Akkermansia* has been found to be the dominant genus in the gut microbiota of Tibetan antelopes and lizards at high altitudes (27). The results of the present study showed that the relative abundance of *Akkermansia* in the intestine of rats in the hypoxia-treated group was significantly reduced, which led to the speculation that the hypoxic environment in the plateau might lead to impaired intestinal barrier integrity and reduced intestinal mucus in rats, which in turn reduced *Akkermansia* transplantation. Interestingly, we also observed that a high-altitude hypoxic environment could significantly increase the abundances of *Clostridium* and *Alistipes*, which are enteric pathogens. These pathogenic bacteria, known as bacterial drivers, elicit an inflammatory process in the gut, thereby accelerating the process of altitude sickness. In the study by Zhang et al. (13), hypoxia-treated mice showed a significant increase in the relative abundance of *Alistipes*, which is consistent with our findings. This suggests that *Alistipes* might be involved in the initiation and development of hypoxia-induced diseases.

Studies on gut microbial function have added another dimension to the characterization of the differences between the gut microbiota of rats raised at different altitudes. Exposure to high-altitude hypoxic environments is a common environmental stress that is associated with physiological and metabolic processes. The analysis of changes in microbial function owing to exposure to high-altitude hypoxic environments demonstrated that the influence of such environments on microbial communities was related to several metabolic pathways, including carbohydrate metabolism, energy metabolism, glycan biosynthesis, and metabolism. This observation further indicated that hypoxia could modify the microbial imprint and subsequently cause physiological and metabolic dysfunction. Westerterp (28) reported that energy imbalance is related to a decrease in food intake, and in hypoxic environments, where metabolic demands exceed energy supply, the body attempts to deal with acute mountain sickness and other diseases, resulting in an energy imbalance. Their analysis also revealed that the genes involved in environmental information processing, cellular processes, and drug development undergo changes in high-altitude hypoxic environments. Similarly, our results demonstrated that the pharmacokinetic characteristics as well as the activity and expression of drug-metabolizing enzymes and transporters significantly change in high-altitude hypoxic environments (29, 30). This offers the possibility for future studies on the mechanism of gut microbiota-mediated drug metabolism in high-altitude hypoxia environments.

Exploring the specific effects of high-altitude hypoxic conditions on the gut microbiota is still associated with several challenges. First, in this study, we used rats as animal models, which are associated with several limitations. Specifically, experimental rats and humans show several differences with respect to gut microbiota structure and diversity. Second, experimental animals cannot fully and objectively reflect the actual characteristics of the human body. Further, to verify whether the gut microbiota is affected by hypoxia, we cleared the gut microbiota of rats using broad-spectrum antibiotic treatment to establish the FMT model. However, germfree rats are more suitable for studying the effect of high-altitude hypoxia on the gut microbiota than pseudogermfree rats treated with antibiotics. In addition to the low oxygen levels in the plateau environment, whether strong radiation, cold, and other factors affect the composition and function of the gut microbiota remains unclear. Gut microbiota complexity makes the study of changes in gut microbiota composition under high-altitude conditions more challenging. Therefore, the factors that can ultimately affect gut microbiota composition and function to bring about changes in behavioral phenotypes and physiological characteristics are yet to be comprehensively explored.

This study showed that a high-altitude hypoxic environment has a considerable effect on gut microbiota. Importantly, the FMT experiment further indicated that plateau hypoxia exerts a significant impact on the gut microbiota, and such changes in the gut microbiota might be related to the plateau hypoxia adaptation process. Based on these results, we suggested that the use of FMT as treatment strategy for some diseases caused by high-altitude hypoxic environments must be further explored. Overall, our results indicated that hypoxic environments exert an impact on the gut microbiota, providing novel insights into the study of drug metabolism in the unique environment of high-altitude hypoxia.

In conclusion, different gut microbiota characteristics, including α-diversity, β-diversity, composition at different taxonomic levels, and bacterial function in different high-altitude hypoxic environments, were analyzed. According to the obtained results, altitude can cause changes in gut microbiota structure and diversity, and this could be attributed to exposure to different altitudes and hypoxia treatment times. Therefore, this study established a foundation for further research on the initiation and development of diseases, as well as drug metabolism, under high-altitude hypoxia.

## MATERIALS AND METHODS

### Experimental design.

To investigate the relationship between a high-altitude hypoxic environment and gut microbiota, rats were housed at a normal altitude, moderate altitude, and high altitude. Fecal samples were collected at different time points and subjected to 16S rRNA gene sequencing. FMT experiments were also performed to further verify the effect of hypoxia on gut microbiota.

### Chemicals and reagents.

Vancomycin (lot C11498870), neomycin sulfate (lot C11606396), metronidazole (lot C111653035), and ampicillin (lot C10868057) were purchased from Shanghai Macklin Biochemical Co. Ltd. (Shanghai, China). HiPure stool DNA kits (lot D3141) were obtained from Guangzhou Magen Biological Technology Co. Ltd. (Guangzhou, China). Q5 high-fidelity DNA polymerase was obtained from New England Biolabs, Inc. (NEB; Ipswich, MA, USA). A NanoDrop 2000 microspectrophotometer was obtained from Thermo Fisher Scientific (Waltham, MA, USA), the ABI StepOnePlus real-time PCR system was obtained from Life Technologies (Foster City, CA, USA), and a Qubit 3.0 fluorimeter was acquired from Thermo Fisher Scientific.

### Animals and FMT.

This study was approved by the Ethical Committee of Qinghai University (approval number 2017-15), and all the experiments were performed in strict compliance with the Guide for the Care and Use of Medical Laboratory Animals of the Ethics Committee of Qinghai University, Xining, China (31). Male Sprague-Dawley specific-pathogen-free (SPF) inbred rats (180 ± 20 g) were obtained from the Experimental Animal Center of Xi’an Jiaotong University (Xi’an, China) (license number SCXX [Shaanxi] 2018-001). All rats were maintained in a temperature (23°C ± 2°C)-controlled and humidity (55% ± 5%)-controlled room with a 12-h light/dark cycle. The rats were allowed to acclimatize to the environment for 1 week prior to experiments, with access to irradiated standard chow and acidified water every day.

The rats were randomly divided into the plain group (P; altitude, 390 m; PaO_2_, 20 kPa), moderate-altitude hypoxic group (M; altitude, 2,800 m; PaO_2_, 15.1 kPa), and high-altitude hypoxic group (H; altitude, 4,300 m; PaO_2_, 12.4 kPa). The rats in the P group (*n* = 6) were bred in the city of Xi’an, located northwest of China’s Shaanxi Province, while those in the M and H groups were transported by bus to Gonghe and Maduo counties, respectively, in Qinghai Province, China. The rats in the M group were further divided as follows: the M3 group was subjected to hypoxic treatment for 3 days, the M7 group was subjected to hypoxic treatment for 7 days, the M15 group was subjected to hypoxic treatment for 15 days, and the M30 group was subjected to hypoxic treatment for 30 days, with six rats in each group. Meanwhile, the rats in the H group were further divided as follows: the H3 group was subjected to hypoxic treatment for 3 days, the H7 group was subjected to hypoxic treatment for 7 days, the H15 group was subjected to hypoxic treatment for 15 days, and the H30 group was subjected to hypoxic treatment for 30 days, with six rats in each group.

Another group of rats was used for FMT experiments. Briefly, the recipient groups comprised normal rats receiving autoclaved saline (FMT-P), a fecal suspension from the 7th-day moderate-altitude hypoxic donor rats (FMT-MH), and a fecal suspension from the 7th-day high-altitude hypoxic donor rats (FMT-HH) (*n* = 6). The rats in the recipient groups were administered an antibiotic cocktail (vancomycin, 0.5 g/L; neomycin sulfate, 1 g/L; ampicillin, 1 g/L; and metronidazole, 1 g/L) to deplete their gut microbiota. This antibiotic treatment was continued for 7 days before the FMT treatment. For FMT, fecal samples from the M7 and H7 donor rats were freshly collected and pooled in equal amounts within each group. Thereafter, 1 g of the pooled fecal samples from each donor group was suspended in 10 mL of sterile 0.9% autoclaved saline via vortexing. FMT was then performed via the oral administration of 10 mL/kg of fecal suspension for 1 week.

Fecal microbiota samples were collected from the individual rats and immediately stored at −80°C for subsequent analysis.

### Analysis of gut microbiota via 16S rRNA gene sequencing.

Total bacterial DNA was extracted from each fecal sample using the HiPure stool DNA kit, and further analysis was conducted by Gene Denovo Technology Co., Ltd. The V3-V4 region of the rRNA gene was amplified via PCR (98°C for 30 s, followed by 30 cycles at 98°C for 10 s, 60°C for 30 s, and 72°C for 30 s and a final extension at 72°C for 120 s) using primers 341F (5′-CCTACGGGNGGCWGCAG-3′) and 806R (5′-GGACTACHVGGGTATCTAAT-3′). The amplicons were purified using the AxyPrep DNA gel extraction kit and quantified using the ABI StepOnePlus real-time PCR system before sequencing on the Illumina platform.

### 16S rRNA sequence data analyses.

The raw reads were processed according to the following criteria. (i) Reads containing more than 10% unknown nucleotides and less than 50% bases with a quality score (*q* value) of >20 were removed. (ii) Paired-end clean reads were merged as raw tags with a minimum overlap of 10 bp and mismatch error rates of 2%. (iii) Raw tags were removed from the first low-quality base site where the number of bases in the continuous low-quality value (the default quality threshold is ≤3) reached the set length (the default length was 3 bp), and tags whose continuous high-quality base length was <75% of the tag length were filtered. The clean tags were clustered into operational taxonomic units (OTUs) of ≥97% similarity using the UPARSE (version 9.2.64) pipeline. All chimeric tags were removed using the UCHIME algorithm, and effective tags were obtained for further analysis. The taxonomy of each 16S rRNA gene sequence was analyzed using RDP classifier software (version 2.2) against the Silva database (version 132), with a confidence threshold value of 0.8. The α-diversity indices, including Shannon, Good’s coverage, and Pielou’s evenness index, were calculated using QIIME (version 1.9.1). The β-diversity indices (PCoA) were generated in the R Project Vegan package (version 2.5.3) and plotted in the R Project ggplot2 package (version 2.2.1). The stacked bar plot of the community composition was visualized in the R Project ggplot2 package (version 2.2.1). Biomarker features in each group were screened using the labdsv package (version 2.0-1) and the R Project randomForest package (version 4.6.12). A ternary plot of species abundance was constructed using the R ggtern package (version 3.1.0). The KEGG pathway analysis of the OTUs was inferred using Tax4Fun (version 1.0). Microbiome phenotypes of bacteria were classified using BugBase.

### Statistical analysis.

Differences between the various groups in terms of α-diversity indices were assessed by performing Kruskal-Wallis rank sum tests. PCoA based on the abundances of each sample was performed to evaluate the degree of similarity between the samples. The random-forest model was applied to classify different treatments. The differences were considered statistically significant at a *P* value of <0.05. The data sets were analyzed using R software (version 3.5.1).

### Data availability.

The raw reads from amplicon sequencing were deposited in the NCBI Sequence Read Archive database (BioProject number PRJNA830832).
